# Increased serum VEGF and b-FGF in Graves’ ophthalmopathy

**DOI:** 10.1007/s00417-014-2662-y

**Published:** 2014-05-28

**Authors:** Xiaozhen Ye, Jun Liu, Yangtian Wang, Lu Bin, Jian Wang

**Affiliations:** Department of Endocrinology, Nanjing Jinling Hospital, Nanjing, 210002 China

**Keywords:** Vascular endothelial growth factor, Basic fibroblast growth factor, Graves’ ophthalmopathy, Growth factors, Inflammatory disorder

## Abstract

**Background:**

Graves’ ophthalmopathy (GO) is thought to be an inflammatory disorder of autoimmune background. The aim of this study is to investigate the involvement of vascular endothelial growth factor (VEGF) and basic fibroblast growth factor (b-FGF) in patients with Graves’ ophthalmopathy (GO).

**Methods:**

Serum concentrations of VEGF and b-FGF of 48 GO patients, 30 Graves’ hyperthyroid disease (GD) patients without ophthalmopathy, and 30 healthy controls were measured by Enzyme-Linked Immunosorbent Assay (ELISA). Patients with GO were subdivided into two groups according to clinical activity scores (CAS): a score of 3 or less is considered as inactive (CAS ≤ 3, inactive GO, *n* = 14), and 4 or more is considered active eye disease (CAS ≥ 4, active GO; *n* = 34). All of the patients with active GO underwent corticosteroid therapy.

**Results:**

The concentrations of serum VEGF and b-FGF were significantly higher in patients with GO and in those with GD than in controls. The serum levels of VEGF and b-FGF in patients with active GO were higher than those in patients with inactive GO and those in GD patients (*P* < 0.05). Moreover, serum VEGF and b-FGF concentratison were significantly correlated with CAS in GO patients (*p* < 0.01). Mean VEGF and b-FGF levels in corticosteroid-responsive patients (CAS decreases ≥3 after treatment) decreased significantly after corticosteroid treatment (*P* < 0.05), and these changes were accompanied by a decrease of CAS (*P* < 0.05).

**Conclusion:**

The results suggest that serum VEGF and b-FGF levels were increased in patients with active GO and could reflect the degree of ocular inflammatory activity.

## Introduction

Graves’ ophthalmopathy (GO), also known as thyroid-associated ophthalmopathy (TAO), is thought to be an inflammatory disorder of autoimmune background, although the pathophysiology of GO remains unclear. GO manifests as orbital inflammation and expansion of fat and extraocular muscles. Orbital T cells, fibroblasts, adipocytes, and perhaps other residential cells release numerous cytokines, growth factors including Vascular endothelial growth factor (VEGF), basic fibroblast growth factor (b-FGF), and inflammatory mediators, many of which act as potent stimulators of glycosaminoglycan accumulation and edema formation [1].

VEGF and b-FGF are potent angiogenic factors whose activities include endothelial cell and fibroblast survival, proliferation, migration, and tube formation [2]. VEGF and b-FGF are critical mediators of physiological as well as pathological angiogenesis. Increased b-FGF and VEGF levels have been reported in several inflammatory diseases [3, 4]. During inflammation, the presence of VEGF and b-FGF has been associated with more intense accumulation of leukocytes and exacerbation of injury [5–7]. Zittermann SI et al. [8] demonstrated that b-FGF has direct and acute effects on normal tissue that synergistically enhance recruitment of polymorphonuclear leucocytes, monocytes, and T cells in response to inflammatory cytokines and delayed type hypersensitivity reactions, independent of angiogenesis. They also reported VEGF and b-FGF differentially enhance monocyte and neutrophil recruitment to inflammation [9]. More recently, Matos et al. [10] has found a positive immunohistochemical expression of VEGF and b-FGF in extraocular rectus, orbital fibrous, and adipose tissue of GO patients. Thus, this study was further carried out to investigate the serum VEGF and b-FGF levels in GO patients and evaluate the association between the serum concentrations of VEGF and b-FGF and the degree of ocular inflammatory activity of GO.

## Subjects and methods

The study was carried out in three groups of subjects. One group had 48 patients with clinical symptoms of ophthalmopathy (GO). Patients who had had prior treatment with steroids or radiation were excluded. Ophthalmopathy was diagnosed by performing a complete eye examination, and the clinical activity of GO was scored according to the clinical activity score (CAS) suggested by Mourits et al. [11] (Table 1). The extraocular muscle (EOM) involvement was evaluated as positive when the patient had diplopia on a binocular single vision test and corresponding extraocular muscle enlargement on the computerized tomography (CT) scan. According to CAS, patients with GO were subdivided into two groups: a score of 3 or less was considered inactive (CAS ≤ 3, inactive GO, *n* = 14), and 4 or more was considered active eye disease (CAS ≥ 4, active GO; *n* = 34). The clinical severity of active GO was scored according to the ophthalmopathy index (OI) based on the NOSPECS classification. All the patients with active GO underwent corticosteroid therapy consisting of intravenous infusions of methylprednisolone (MP) in two series of 3 g each session for 2 weeks and subsequent treatment with oral prednisone (P) at 60 mg per day for two months and then a gradual tapering schedule with a reduction of 5 mg per week by 14 weeks). The serum samples were collected 24 h before MP and at the end of corticosteroid therapy. The second group had 30 patients with Graves’ disease (GD) without symptoms of ophthalmopathy. Both GO and GD patients were euthyroid before the study. Euthyreosis was confirmed by thyrotropin and free thyroxine estimation. All of the patients of both of these groups were treated with thiamazol. None had any other autoimmune-related disease. The third group had 30 healthy volunteers (control group) age-matched and sex-matched to group 1 and 2 who had neither family history of Graves’ disease nor other autoimmune diseases. No acute infections were observed in the GO, GD, and control subjects three weeks prior to the study. In all cases, a written informed consent was obtained from the patients. This study was approved by the Local Hospital Bioethical Committee.

**Table 1:** The 10 items of the clinical activity score (CAS)

Pain	1 Painful, oppressive feeling on or behind the globe during the last 4 weeks
	2 Pain on attempted up, side, or down gaze during the last 4 weeks
Redness	3 Redness of the eyelid(s)
	4 Diffuse redness of the conjunctiva, covering at least one quadrant
Swelling	5 Swelling of the eyelid(s)
	6 Chemosis
	7 Swollen caruncle
	8 Increase of proptosis of ≥2 mm during a period of 1–3 months
Impaired function	9 Decrease of eye movements in any direction ≥5° during a period of 1–3 months
	10 Decrease of visual acuity of ≥1 line(s) on the Snellen chart (using a pinhole) during a period of 1–3 months

For each item present, one point is given. The sum of these points is the CAS

All the sera samples were obtained at 8:00 AM after an overnight fast and kept frozen at −80°C until used. The levels of serum b-FGF and VEGF were determined by the ELISA method (Parameter kit, Bender Med Systems, Vienna, Austria): b-FGF (sensitivity 30pg/ml; intra-assay precision 10%, inter-assay precision 10%) and VEGF (sensitivity 14pg/ml; intra-assay precision 6.8%, inter-assay precision 8.3%). The levels of serum TSH receptor antibody (TRAB) were also measured.

The statistical package SPSS 13 (SPSS Inc, Chicago, IL, USA) was used for analysis. Values were expressed as Mean ± Standard Deviation (SD). One-way ANOVA (adjusted by Bonferroni correction) was applied for multiple comparisons of data, while the Student’s *t* test was used for pairwise comparisons. The correlation of CAS with b-FGF and VEGF levels were evaluated with a Spearman correlation test. Statistical significance was defined as *P* < 0.05.

## Results

### GO patients, GD patients, and control subjects

Thirty-four patients with active GO, 14 patients with inactive GO, 30 GD patients without ophthalmopathy but with duration of disease from 3–24 months, and 30 healthy control subjects were studied. Their age, sex, CAS scores, ophthalmopathy duration (the time from eye disease symptom onset to sampling for each of the groups), exophthalmometry, TRAb, and OI are summarized in Table 2. There was no statistical difference in age or sex between the groups.

**Table 2:** Characteristics of GO patients, GD patients, and control participants (mean ± SD)

	Active GO	Inactive GO	GD	Normal control	P value
N	34	14	30	30	
Sex (female:male)	23:11	9:5	19:11	20:10	0.62
Age (years)	31.06 ± 15.15	30.79 ± 17.80	34.50 ± 13.45	32.8 ± 10.8	0.60
Ophthalmopathy duration	10.06 ± 7.16	12.64 ± 10.78			0.88
CAS	5.65 ± 1.72	2.57 ± 0.65	NA	NA	<0.001
TSH (μIU/ml)	0.94 ± 2.46	1.48 ± 2.67	1.23 ± 2.07	NA	0.34
Free T4 (ng/dl)	1.61 ± 0.92	1.52 ± 0.87	1.45 ± 0.79	NA	0.72
Exophthalmos (mm)	19.5 ± 2.76	17.31 ± 2.51	NA	NA	0.03
OI	8.35 ± 1.28	5.71 ± 1.54	NA	NA	0.000
TRAb	638.37 ± 261.34	423.03 ± 190.94	356.11 ± 160.56	NA	0.008

*NA* not applicable

### Increased serum concentration of b-FGF and VEGF in GO

The levels of serum b-FGF and VEGF are shown in Table 3. We found a significantly higher level of b-FGF and VEGF in the GD and GO groups than in the control group (*P* < 0.05). The serum levels of b-FGF and VEGF in GO were higher than in GD (*P* < 0.05); the serum levels of b-FGF and VEGF in active GO cases were higher than those in inactive GO cases and in GD cases (*P* < 0.05). However, the serum concentrations of b-FGF and VEGF were not significantly different between inactive GO cases and GD cases (*P* > 0.05).

**Table 3:** Serum b-FGF and VEGF concentrations in GO patients (active GO and inactive GO), GD patients, and the control group (NC) (mean ± SD)

Growth factors	Active GO (*n* = 34)	Inactive GO (*n* = 14)	GD (*n* = 30)	Normal control (*n* = 30)
VEGF(pg/ml)	182.76 ± 75.17^c^ ^e^	132.34 ± 42.19^b^	125.46 ± 34.82^c d^	76.45 ± 6.81^f^
b-FGF(pg/ml)	101.52 ± 35.53^c^ ^e^	78.58 ± 17.37^a^	75.38 ± 13.53^b d^	63.71 ± 7.40^e^

–vs. controls, ^a^ *P* < 0.05, ^b^ *P* < 0.01, ^c^ *P* < 0.001;

–vs. Inactive GO, ^d^ *P* > 0.05, ^e^ *P* < 0.05, ^f^ *P* < 0.01;

### Serum concentration of b-FGF and VEGF in patients with active GO after corticosteroid treatment

The serum b-FGF and VEGF levels during corticosteroid treatment in patients with active GO are shown in Table 4. A CAS change greater or equal to 3 after treatment is considered as corticosteroid-responsive, and a change of less than 3 was considered corticosteroid-resistant. There were significant differences in serum b-FGF and VEGF levels between the corticosteroid-responsive patients (*n* = 25) and the corticosteroid-resistant individuals (*n* = 9). The pre-treatment b-FGF and VEGF levels were significantly elevated in the corticosteroid-responsive patients compared with the corticosteroid-resistant subjects (*P* < 0.05). In corticosteroid-responsive patients, b-FGF and VEGF levels were significantly decreased after the treatment (*P* < 0.05), and these changes were accompanied by a decrease of CAS (*P* < 0.05). There were no differences in the post-treatment b-FGF and VEGF levels between the corticosteroid-responsive patients and the corticosteroid-resistant subjects (*P* > 0.05). In corticosteroid-resistant patients, the pre-treatment b-FGF and VEGF levels were higher than in patients with inactive GO (*P* < 0.05).

**Table 4:** Serum b-FGF and VEGF levels in patients with active GO before and after corticosteroid treatment (mean ± SD)

		Before corticosteroid treatment	After corticosteroid treatment
b-FGF (pg/ml)	Corticosteroid-responsive (*n* = 25)	124.61 ± 38.30^a^ ^c e^	78.00 ± 14.19^d^
	Corticosteroid-resistant (*n* = 9)	100.23 ± 41.62^b e^	91.07 ± 38.54
	Inactive GO (*n* = 14)	78.58 ± 17.37	
VEGF (pg/ml)	Corticosteroid-responsive (*n* = 25)	250.58 ± 71.69^a c e^	133.46 ± 34.97^d^
	Corticosteroid-resistant (*n* = 9)	192.58 ± 53.24^b e^	163.57 ± 44.79
	Inactive GO (*n* = 14)	132.34 ± 42.19	
CAS	Corticosteroid-responsive (*n* = 25)	7.50 ± 2.61^a c e^	2.54 ± 1.12^d^
	Corticosteroid-resistant (*n* = 9)	4.08 ± 1.98^b e^	3.21 ± 1.56
	Inactive GO (*n* = 14)	2.57 ± 0.65	
OI	Corticosteroid-responsive (*n* = 25)	9.75 ± 1.83^a c e^	4.63 ± 1.02
	Corticosteroid-resistant (*n* = 9)	7.82 ± 1.13^b e^	4.51 ± 1.18
	Inactive GO (*n* = 14)	5.71 ± 1.54	

–vs. post-treatment values, ^a^ *P* < 0.05, ^b^ *P* > 0.05;

–vs. corticosteroid-resistance, ^c^ *P* < 0.05, ^d^ *P* > 0.05;

–vs. Inactive GO, ^e^ *P* < 0.05, ^f^ *P* > 0.05;

### Correlation between disease activity and growth factors

**Fig. 1:**
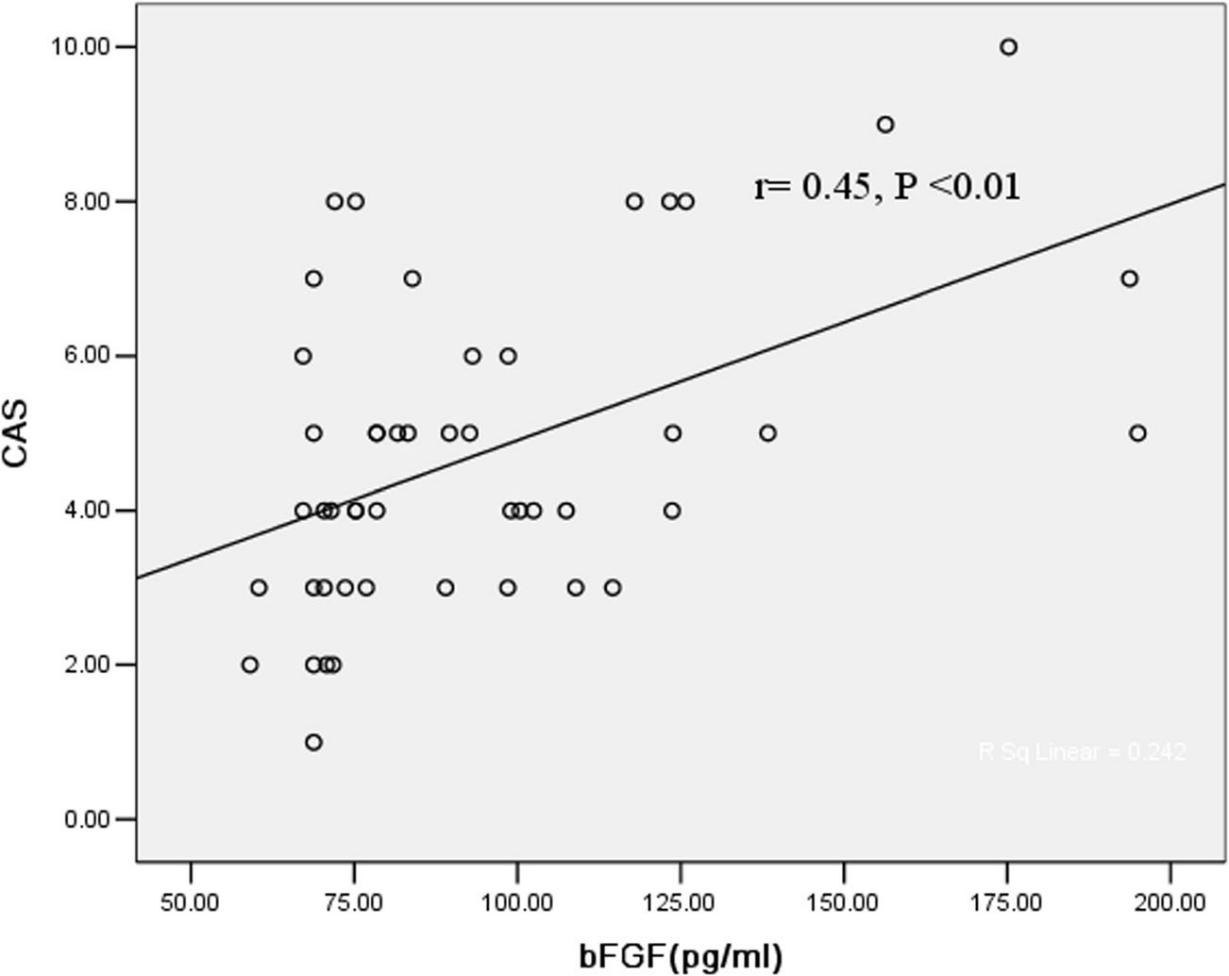
Correlation between clinical activity score (CAS) and serum b-FGF

**Fig. 2:**
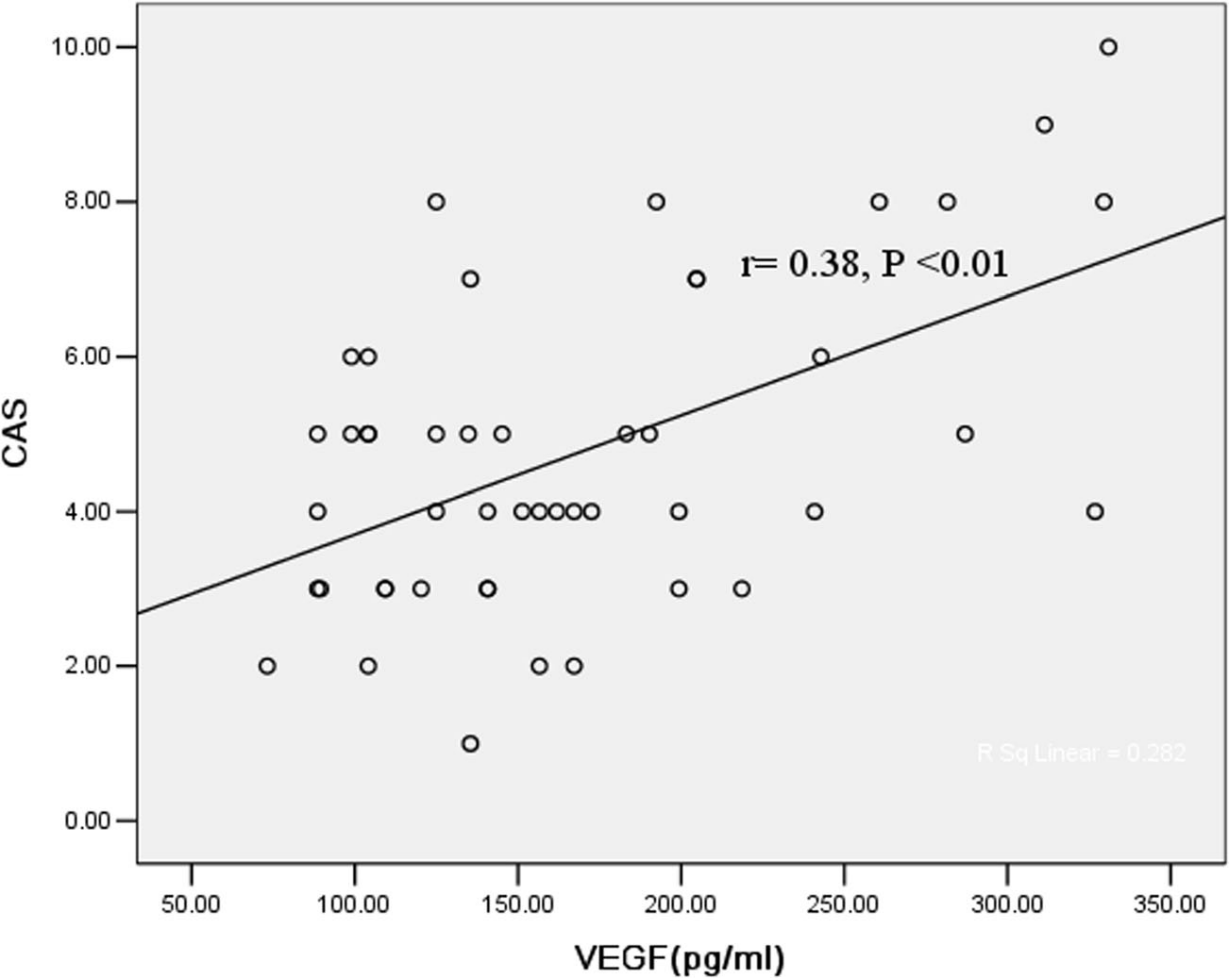
Correlation between clinical activity score (CAS) and serum VEGF

There were significant correlations of CAS with exophthalmometry (*p* < 0.001) in patients with either active or inactive GO. The positive correlation of CAS with b-FGF in GO patients was shown in Fig. 1. Serum VEGF concentration had a significant correlation with CAS in GO patients (*p* < 0.01) (Fig. 2). Moreover, significant correlations between b-FGF and VEGF (*r* = 0.61, *p* < 0.05) were noted. The elevated levels of bFGF, VEGF, and CAS were all positively correlated with TRAb (*r*1 = 0.37, *p* < 0.01; *r*2 = 0.27, *p* < 0.05; *r*3 = 0.72, *p* < 0.01).

## Discussion

GO is an autoimmune condition characterized by infiltration of the extraocular muscles (EOM) and/or the orbital fat/connective tissue by lymphocytes and other mononuclear cells. Recruitment of leukocytes from the blood into orbit is a critical process in inflammation and immune responses. Many diseases characterized by leukocyte infiltration are associated with both angiogenesis and the presence of b-FGF and VEGF in the tissue [12].

We attempted to determine whether serum VEGF and b-FGF increase in GO, which correlates with disease activity. The sequential increase of VEGF and b-FGF concentrations in the normal group, the GD patient group, or inactive GO patient group, and the active GO patient group suggests that VEGF and b-FGF may play a role in GO through the recruitment of the effector leukocytes into the inflamed tissue sites, thus orchestrating the immune response at the site of inflammation. The CAS in GO was significantly correlated with serum VEGF and b-FGF concentrations. Moreover, b-FGF and VEGF levels were significantly decreased in corticosteroid-responsive patients after corticosteroid treatment, and these changes were accompanied by a decrease of CAS. Thus, our data further suggest that VEGF and b-FGF may have a relevant role in the inflammatory phenomenon seen in GO.

b-FGF, produced by endothelial cells and fibroblasts, is one of the most potent stimulators of angiogenesis and is mitogenic and chemotactic for both endothelial cells and fibroblasts [13–15]. Inflammatory cytokines have been reported to induce b-FGF synthesis [16, 17]. The expression and secretion of b-FGF is increased at sites of inflammation. b-FGF is increased in the serum and affected tissue of patients with rheumatoid arthritis, inflammatory bowel disease, or asthma [18–21]. Zittermann SI et al. [22] demonstrated that b-FGF increases the recruitment of monocytes, T cells, and polymorphonuclear neutrophils to inflammatory dermal sites. Strong immunoexpression of b-FGF has been found in the orbit fibroblasts of patients with GO [10]. In this study, the serum concentration of b-FGF was found to significantly increase in GO patients, especially in those with active GO. We consider that this release could occur because of orbit fibroblast damage. This local release of b-FGF may normally lead to fibroblast proliferation and secretion of collagen and glycosaminoglycans.

VEGF is produced by endothelial cells, macrophages, activated T cells, and a variety of other cell types [2]. It was originally identified as a potent enhancer of vascular permeability [23], and this could account for the increased leukocyte recruitment [24]. Ito [25] reported the presence of VEGF mRNA-expressing cells scattered in edematous stroma, suggesting that plasma cells may play an important role in the development of edema in chronic inflammation via the production of VEGF. Overexpression of VEGF mRNA by infiltrating mononuclear cells has been found in delayed hypersensitivity reactions [26]. Reports also suggest that VEGF acts as an angiogenic mediator in rheumatoid arthritis [27, 28]. Strong immunoexpression of VEGF has been found in the orbit endothelial cells of patients with GO [10]. Figueroa et al. [29] found that levels of VEGF tended to be higher in patients with active GO (*n* = 13) compared with GD patients (*n* = 18) and patients with inactive GO (*n* = 13), though no significant differences were reached.The present study with a higher number of patients was further conducted. On the basis of the significantly increased VEGF levels in the patients with GO, VEGF may be expected to play an important role in the regulation of orbital inflammation in GO.

In conclusion, we demonstrated that the serum VEGF and b-FGF levels were elevated in patients with increased inflammatory signs, and that successful management of active GO with corticosteroids was associated with a decrease in these two growth factors. Serum VEGF and b-FGF levels might play a role in the acute phase of inflammation and could reflect the degree of ocular inflammatory activity in GO patients.
